# Protein and lipid MALDI profiles classify breast cancers according to the intrinsic subtype

**DOI:** 10.1186/1471-2407-11-465

**Published:** 2011-10-27

**Authors:** Han Sung Kang, Seok Cheol Lee, Young Seung Park, Young Eun Jeon, Jeong Hwa Lee, So-Youn Jung, In Hae Park, Seok Hoon Jang, Hye Min Park, Chong Woo Yoo, Seok Hee Park, Sang Yun Han, Kwang Pyo Kim, Young Hwan Kim, Jungsil Ro, Hark Kyun Kim

**Affiliations:** 1 National Cancer Center, Goyang, 410-769, Korea; 2 Division of Mass Spectrometry Research, Korea Basic Science Institute, Ochang, 363-883, Korea; 3 Center for Nano-Bio Convergence, Korea Research Institute of Standards and Science, Daejeon, 305-340, Korea; 4 Department of Molecular Biotechnology, WCU Program, Konkuk University, Seoul, 143-701, Korea; 5 Department of Biological Sciences, Sungkyunkwan University, Suwon, 440-746, Korea; 6 Graduate School of Analytical Science and Technology, Chungnam National University, Daejeon, 305-764, Korea

**Keywords:** protein, lipid, breast cancer, MALDI

## Abstract

**Background:**

Matrix-assisted laser desorption/ionization (MALDI) mass spectrometry (MS) has been demonstrated to be useful for molecular profiling of common solid tumors. Using recently developed MALDI matrices for lipid profiling, we evaluated whether direct tissue MALDI MS analysis on proteins and lipids may classify human breast cancer samples according to the intrinsic subtype.

**Methods:**

Thirty-four pairs of frozen, resected breast cancer and adjacent normal tissue samples were analyzed using histology-directed, MALDI MS analysis. Sinapinic acid and 2,5-dihydroxybenzoic acid/α-cyano-4-hydroxycinnamic acid were manually deposited on areas of each tissue section enriched in epithelial cells to identify lipid profiles, and mass spectra were acquired using a MALDI-time of flight instrument.

**Results:**

Protein and lipid profiles distinguish cancer from adjacent normal tissue samples with the median prediction accuracy of 94.1%. Luminal, HER2+, and triple-negative tumors demonstrated different protein and lipid profiles, as evidenced by permutation *P *values less than 0.01 for 0.632+ bootstrap cross-validated misclassification rates with all classifiers tested. Discriminatory proteins and lipids were useful for classifying tumors according to the intrinsic subtype with median prediction accuracies of 80.0-81.3% in random test sets.

**Conclusions:**

Protein and lipid profiles accurately distinguish tumor from adjacent normal tissue and classify breast cancers according to the intrinsic subtype.

## Background

Proteomics research is actively being performed to find biomarkers for common solid tumors [1,2] including breast cancer [3-5]. Matrix-assisted laser desorption/ionization (MALDI) mass spectrometry (MS) has been demonstrated to be useful for histological classification [6-10] and outcome prediction [11] of common solid tumors.

In this approach, thin sections of frozen tissues are obtained from surgical resections or biopsies and mass spectra are obtained from discrete locations on the tissue. In a study of human breast cancer samples, protein profiles obtained from histology-directed MALDI MS differentiate invasive breast cancers from ductal carcinoma in situ and normal breast epithelium [12]. In another report, MALDI imaging MS classified breast cancer tissue specimens according to HER2 status [13].

Accumulating evidence suggests that alteration in lipid composition is associated with breast carcinogenesis [14-16]. Recently, cancer-associated lipid alteration was extensively characterized using ultra performance liquid chromatography-MS analysis of tissue lysate from breast cancer tissue specimens [16]. These reports suggest that monitoring lipid composition in clinical samples may provide an opportunity for breast cancer diagnosis.

Breast cancer is the second most commonly diagnosed cancer in women worldwide [17]. Recent advances in the development of matrices for MALDI MS made it possible to directly probe tissues to profile lipid composition and distribution [18,19]. Using frozen surgical breast cancer tissue samples, we performed a comprehensive, histology-directed MALDI MS analysis of protein and lipid to evaluate whether this approach can differentiate and classify breast cancers. Here we demonstrate that protein and lipid MALDI MS profiles accurately differentiate breast cancers from normal epithelium, and classify breast tumors according to their intrinsic subtype.

## Methods

### Collecting and processing clinical material and protein MALDI MS analysis

Thirty-four pairs of breast cancer and adjacent normal tissue samples were collected at the time of surgery, with informed consent and institutional review board approval, from breast cancer patients undergoing surgery at National Cancer Center in Korea from 2001 to 2010, and stored in liquid nitrogen until analysis. Eleven samples were excluded by spectra quality filter of ClinProTools (version 2.2, Bruker Daltonics). Additional 21 samples were excluded because of inadequate (< 50%) tumor content. These excluded samples were not different from samples analyzed in this study in patient age or intrinsic subtype. Breast cancer intrinsic subtypes were classified according to an immunohistochemistry (IHC) surrogate panel [20,21] as follows: luminal [estrogen receptor (ER) positive and/or progesterone receptor (PR) positive], HER2+ [HER2+ regardless of hormone receptor status], and triple-negative [ER-, PR-, and HER2-]. A cut-off value of 1% or more of positively stained nuclei was used to define ER and PR positivity. HER2 was scored as 0-3+ according to the method recommended for the HercepTest (Dako, Glostrup, Denmark). The cases with IHC scores of 3+ or ERBB2 gene amplification by fluorescence in situ hybridization (FISH) were considered positive for HER2.

Thin (10 μm) sections were obtained from the frozen tissues using a cryostat (Leica CM 3050S, Leica Microsystems Inc., Bannockburn, IL). Multiple (three to seven) serial sections were obtained from each tissue. One section was affixed to a standard glass slide, and then stained with hematoxylin and eosin (H&E). The other sections were thaw-mounted onto an indium tin oxide (ITO) slide (HST Inc., Newark, NJ), desiccated in vacuum for 20 min, and washed with graded ethanol solutions (70%, 90%, and 95% ethanol for 30 sec each) for subsequent protein MALDI MS analysis. Sinapinic acid (SA) was used as the protein MALDI matrix and prepared as a 20 mg/ml solution in 50:50 acetonitrile: 0.1% trifluoroacetic acid (TFA). Using a micropipette, 250 nl of the matrix was manually deposited twice per spot. The H&E-stained serial section was then evaluated by a pathologist, who confirmed that entire H&E section of tumor samples had at least 50% tumor content. Mass spectra were acquired using an Autoflex III (Bruker Daltonics, Bremen, Germany) MALDI TOF equipped with a SmartBeam laser (Nd:YAG, 355 nm) and run using a linear-mode acquisition method optimized for 2-30 kDa, a laser frequency of 200 Hz, and a delay time of 7 ns. A total of 400 spectra were acquired at each spot position.

### Lipid MALDI MS analysis

For lipid MALDI MS analysis, the binary matrix solution was prepared by dissolving 7 mg each of 2, 5-dihydroxybenzoic acid (DHB) and α-cyano-4-hydroxycinnamic acid (CHCA) in 1 mL of 70% methanol plus 0.1% TFA and 1% piperidine [19]. Under the guidance of the same H&E-stained adjacent section, the binary matrix (250 nl × 2) was manually deposited on the tumor-rich area of cryosections that were thaw-mounted onto ITO slides. Mass spectra were acquired both in positive- and negative-ion reflector modes, using an Autoflex III (Bruker Daltonics) TOF equipped with a SmartBeam laser and run using a linear-mode acquisition method optimized for 500-1,200 Da, a laser frequency of 200 Hz (3,000 consecutive laser shots), and a delay time of 0 ns. Before each data acquisition, an external calibration was conducted using lipid-mixed calibration standards with m/z ranges of 674-834 Da (positive ion mode) and 564-906 Da (negative ion mode).

MALDI LIFT (MS/MS) analysis was directly performed on the tissue section after MALDI MS. LIFT data and some lipid databases http://lipidsearch.jp or http://www.lipidmaps.org were used to facilitate and confirm the assignment of phospholipid species.

### Data Processing and Statistical Analysis

ClinProTools was used for baseline subtraction, spectral recalibration, and spectral area calculation. A resolution of 300 was applied to the peak detection method. The Top Hat baseline with 10% minimal baseline width was used for baseline subtraction. Data reduction was performed at a factor of four. Spectra were recalibrated with a maximal peak shift of 2,000 ppm between reference and peak masses. The value of the "% Match to Calibrant Peaks" parameter was set to 20%. Spectra that were not recalibratable were excluded. All data with signal-to-noise ratios > 5 were acquired, and the peak area was used for peak calculation with zero level integration. An average peak list was set up for each tissue sample by choosing peaks on the calculated total average spectrum for each tissue sample to create one average spectrum per patient. Peaks with the highest intensity in each ion mode (m/z = 616.23 and 539.53 in positive and negative ion modes respectively) were identified as non-lipid molecules and therefore excluded from subsequent analysis. Average-normalized datasets (*i.e*., protein, positive mode-lipid, and negative-mode lipid datasets) were then combined into a single dataset and subjected to statistical analysis using BRB-ArrayTools (NCI, version 3.8) [22]. A principal component analysis (PCA) plot was generated using multi-dimensional scaling analysis of BRB-ArrayTools, which graphically represents correlation coefficients among samples without forcing the samples into specific clusters. The three primary principal components were used as the axes for the 3-dimensional scaling representation. Class comparison and class prediction analyses were also performed using BRB-ArrayTools. The class comparison analysis computed the F-test for each peak, and listed peaks differentially expressed among the classes at selected statistical significance level. Then, it performed random permutations of the class labels. For each random permutation, all F-tests were re-computed for each peak. The class comparison tool computed the proportion of the random permutations that gave as many peaks significant at the selected level of significance as were found by comparing the true class labels. Protein and lipid profiles of the classes were considered different if this probability (designated as *Permutation P value*) was calculated < 0.05.

To evaluate whether classes have different protein and lipid profiles, class prediction analyses were performed using all samples as a training set. The 0.632+ bootstrap cross-validated misclassification rates was computed for all classifier functions (compound covariate predictor, diagonal linear discriminant analysis, 1- and 3-nearest neighbors, nearest centroid, and support vector machine) in the training set. Then, class labels were randomly shuffled and the cross-validated misclassification rate was computed for each random dataset. Permutation *P *value, which is defined as the proportion of random datasets that give as small misclassification rate as is obtained with real class labels, was then calculated. MALDI MS profiles of the classes were considered different if this permutation *P *value was < 0.05.

To estimate the predictive power of discriminatory peaks, the class prediction analyses were also performed by randomly dividing the whole sample into two (training and test) subsets at 1-to-1 ratio. Randomization was performed using nQuery Advisor software (version 7.0, Statistical Solutions, Saugus, MA).

## Results

MALDI MS analyses were performed for 34 pairs of retrospective surgical tissue samples (34 breast cancers and 34 adjacent normal tissue samples). Adjacent normal tissue samples were collected at least 2 cm apart from the cancer margin. Table 1 summarizes the clinico-pathological characteristics of analyzed tissue samples. There were 30 adenocarcinomas (luminal (n = 18), HER2+ (n = 7), and triple-negative (n = 5)), and 4 metaplastic carcinomas. In 16 out of 34 breast cancer samples (47.1%), the entire sample had > 50% tumor content. In the remaining 18 samples (52.9%), the pathologist marked the hematoxylin and eosin (H&E) slide at the tumor-rich (> 50% tumor content) area, being careful to deposit the matrix within the boundary of the marked tumor-rich area (Additional File 1, Figure S1).

**Table 1:** Clinico-pathological characteristics

**Age, yr**	
Range	33-73
Median	45
**Gender**	
Female	34 (100%)
**AJCC Stage**	
IA	6 (17.6%)
IIA	16 (47.1%)
IIB	1 (2.9%)
IIIA	9 (26.4%)
IIIB	2 (5.9%)
**Location of primary tumor**	
Unilateral	34 (100%)
Right	18 (52.9%)
Left	16 (47.1%)
Bilateral	0
**Histologic classification**	
Adenocarcinoma	30 (88.2%)
Ductal	26 (86.7%)
Lobular	1 (3.3%)
Mixed ductal and lobular	1 (3.3%)
Mucinous	2 (6.6%)
Metaplastic	4 (11.7%)
**Immunohistochemistry**	
**Estrogen receptor (ER)**	
Positive	24 (70.6%)
Negative	10 (29.4%)
**Progesterone receptor (PR)**	
Positive	26 (76.5%)
Negative	8 (23.5%)
**HER2**	
Positive	14 (41.2%)
Negative	20 (58.8%)
**Ki67**	
< 15%	14 (41.2%)
≥15%	20 (58.8%)
**Intrinsic subtype**	
Adenocarcinoma	30 (88.2%)
Luminal (ER+ and/or PR+)	18 (60.0%)
HER2 (HER2+ regardless of ER status)	7 (23.3%)
Triple negative (ER-, PR-, and HER2-)	5 (16.7%)
Metaplastic	4 (11.7%)

AJCC, American Joint Committee on Cancer (7^th ^Edition)

**Figure 1:**
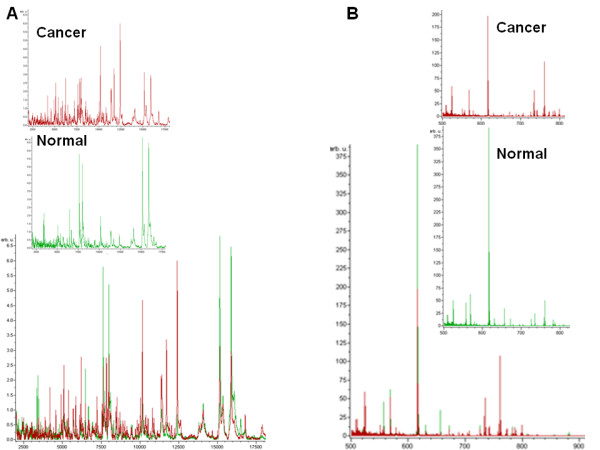
**Average mass spectra for proteins (A) and lipids (B) obtained in the positive ion mode from breast cancer and adjacent normal tissue**. Overlays of cancer and normal tissue mass spectra are shown below.

Mass spectra were acquired on individual spots for each tissue section, and these spectra were averaged together after pre-processing to create one average spectrum per patient. The average spectra were composed of 2 to 11 individual protein measurements for cancer samples (with a median value of 5), and 2 to 10 individual protein measurements for adjacent normal samples (with a median value of 5). The average spectra were composed of 3 to 8 individual lipid measurements (with a median value of 4) for both cancer and normal samples. It was possible that same tissue area was profiled multiple times (up to three spots) from different tissue sections loaded on different ITO slides. Individual measurements were averaged to minimize intra-sample variability. Post-spectral processing identified 85 protein and 144 lipid features (78 for the positive ion mode and 66 for the negative ion mode) across the entire mass range for all of the samples studied (Figure 1).

### Cancer *versus *adjacent normal breast tissue

**Figure 2:**
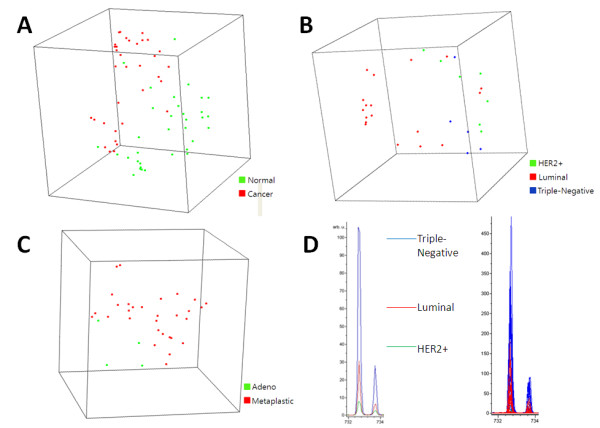
**Principal component analysis and Intensity Profile**. (A) A principal component analysis plot for 34 pairs of breast cancer (*shown in red*) and adjacent normal (*shown in green*) tissue samples, which graphically represents 1-correlation distances among samples. Each sphere represents a single sample, and samples whose protein expression profiles are very similar are shown close together. (B) A principal component analysis plot for luminal (*shown in red*), HER2+ (*shown in green*), and triple-negative breast adenocarcinoma samples (*shown in blue*) (C) A principal component analysis plot for metaplastic carcinomas (*shown in green*) and adenocarcinomas (*shown in red*). (D) Intensity profile for a representative lipid (m/z = 732.58 in the positive ion mode) differentially expressed among intrinsic subtypes. The average peak of the intrinsic subtype (*left*) and all single peaks (*right*) are shown.

A principal component analysis (PCA) plot graphically demonstrated that cancer and adjacent normal samples were separately clustered in an unsupervised analysis (Figure 2A). When a class comparison analysis was performed using BRB-ArrayTools, the proportion of the random permutations that gave as many significant peaks at a feature selection of *P *< 10^-5 ^as were found by comparing the true class labels (cancer *vs *normal) was < 0.001, strongly suggesting that the cancer and normal tissue samples have significantly different protein and lipid profiles (Table 2). Class prediction analysis was subsequently performed after randomly dividing the entire set of samples into two groups at 1-to-1 ratio. At a feature selection *P *< 0.001, the median class prediction accuracy in test sets was 94.1% for all classifiers tested, in 100 random training-to-test partitions. Thus, breast cancer and adjacent normal tissues have clearly different protein and lipid profiles.

**Table 2:** Peaks differentially expressed between cancer and adjacent normal tissue samples at a feature selection *P *< 10^-5^

Overexpressed in cancer				Underexpressed in cancer		
m/z	P	Ratio^1^	Assignment	m/z	p	Ratio^1^
6206.58	1.00E-07	1.6		5150.41	3.50E-06	0.9
7222.53	1.00E-06	1.4		7618.64	< 1E-10	0.6
8514.66	1.10E-06	1.4		9486.03	1.00E-07	0.7
11719.24	< 1E-07	1.8		16089.36	9.90E-06	0.6
12411.2	< 1E-07	2.1				
				n545.56	2.70E-06	0.5
p734.58	3.60E-06	2.0	PC {32:0} [M+H]^+^	n554.49	4.00E-07	0.4
p741.62	< 1E-07	2.1	SM {d18:1/16:0} [M+K] ^+^	n568.56	9.60E-06	0.5
p760.62	2.90E-06	2.0	PC {34:1} [M+H]^+^	n574.61	5.70E-06	0.7
p772.58	< 1E-07	1.8	PC {32:0} [M+K]^+^	n594.5	1.00E-07	0.6
p798.58	< 1E-08	2.1	PC {34:1} [M+K]^+^	n609.74	1.90E-06	0.4
				n651.64	3.40E-06	0.5
n505.37	< 1E-09	5.3		n666.79	7.00E-07	0.5
n605.64	< 1E-10	5.3		n679.13	6.40E-06	0.5
				n878.89	2.50E-06	0.6
				n907.09	7.50E-06	0.5
				n917.48	3.60E-06	0.5
				n997.7	1.80E-06	0.5

^1^Ratio, cancer/normal

p, lipids identified in the positive ion mode

n, lipids identified in the negative ion mode

SM, Sphingomyelin

To evaluate whether patients' age affects cancer-associated protein and lipid alteration, the cancer/normal peak area ratio was compared between older (≥50 years, n = 10) and younger (< 50 years, n = 24) age groups at feature selection P < 0.05, using class comparison algorithm of BRB-ArrayTools. The probability of getting as many significant peaks by chance as was obtained with real class labels, if there are no real differences between class, was 0.78, suggesting that cancer-associated protein and lipid alteration was not significantly different between older and younger age groups.

### Classification according to intrinsic subtype

According to the PCA analysis, cancer samples were clustered according to their intrinsic subtypes (Figure 2B). Class comparison was performed between intrinsic subtypes that were defined by immunohistochemistry (luminal [ER+ and/or PR+] *vs *HER2 [HER2 +] *vs *triple-negative [ER-, PR-, and HER2-]. At a feature selection *P *< 0.001, 19 peaks were significantly different among subtypes (Table 3). The probability of getting at least 19 peaks significantly by chance (at the 0.001 level) with no real differences between the classes was 0.002. For class prediction analysis, all 30 cancer samples were first used as a training set. At a feature selection *P *< 0.001, permutation *P *values for 0.632+ bootstrap cross-validated misclassification rates were < 0.01 for all classifiers tested. These results indicate that breast tumor intrinsic types have different protein and lipid profiles. When these analyses were performed separately in protein and lipid datasets, lipid profiles demonstrated consistently lower permutation *P *values for cross-validated misclassification rate, across feature selection *P *values ranging 0.001 to 0.05 (*data not shown*).

**Table 3:** Peaks differentially expressed among intrinsic subtypes at a feature selection *P *< 0.001

mz	p	Relative peak intensity			Assignment
		Luminal	HER2+	Triple-negative	
p514.32	0.0006	2.2	3.8	2.0	
p515.2	0.0007	1.7	2.8	1.3	
p531.34	0.0002	1.3	2.4	1.1	
p534.25	0.0001	1.8	3.0	1.5	PC {16:1} [M+H]^+^
p535.22	0.0001	1.2	1.7	1.1	
p578.67	0.0000	14.6	5.3	3.1	
p644.33	0.0004	3.6	2.1	1.5	
p678.32	0.0006	4.8	4.6	2.6	
p706.52	0.0003	2.4	2.7	5.7	PC {30:0} [M+H]^+^
p732.58	< 0.001	1.3	2.7	11.0	PC {32:1} [M+H]^+^
n505.37	0.0001	13.2	90.5	78.1	
n527.44	0.0005	4.5	1.6	1.6	
n545.56	< 0.001	5.0	1.5	1.7	
n552.56	0.0003	2.3	1.1	1.1	
n554.49	< 0.001	3.8	1.2	1.2	
n587.67	0.0009	4.3	1.6	1.7	
n589.59	0.0002	3.6	1.4	1.4	
n627.71	0.0008	4.7	1.9	2.1	
n631.89	0.0005	2.5	1.3	1.2	

^1^Ratio, cancer/normal

p, lipids identified in the positive ion mode

n, lipids identified in the negative ion mode

We then planned to estimate the predictive power of discriminatory proteins and lipids in training-to-test partitions, although this analysis is limited by the sample size. At a feature selection *P *< 0.05, the median prediction accuracy in test sets ranged from 80% to 81.3% (80%, 81.3%, 80%, and 80%, for diagonal linear discriminant analysis, 1-nearest neighbor, 3-nearest neighbors, and nearest centroid, respectively), in 100 random training-to-test partitions. Discriminatory peaks are listed in Table 3.

### Metaplastic carcinoma *vs *adenocarcinoma

Metaplastic carcinoma is a unique breast cancer subtype, characterized by distinct morphologic feature and poor prognosis [23]. To further evaluate whether protein and lipid profiles could distinguish biological feature, we compared metaplastic carcinomas (n = 4) with adenocarcinomas (n = 30) for protein and lipid profiles. According to PCA analysis, metaplastic carcinomas were clearly separated from adenocarcinomas (Figure 2C). For class prediction analysis, all 34 cancer samples were used as a training set, given the small number of metaplastic carcinomas. At a feature selection *P *< 0.001, permutation *P *values for 0.632+ bootstrap cross-validated misclassification rates were < 0.05 for all classifiers tested (0.01, < 0.01, < 0.01, < 0.01, 0.01, and < 0.01, for the compound covariate predictor, diagonal linear discriminant analysis, 1-nearest neighbor, 3-nearest neighbors, nearest centroid, and support vector machine, respectively). Although sample size is small, metaplastic carcinomas appear to have unique protein and lipid profiles.

## Discussion

This study demonstrates that histology-directed MALDI MS analysis of lipids and proteins may differentiate between cancerous and normal epithelium and among intrinsic subtypes of breast cancer. Phosphatidylcholines (PCs) {34:1} (m/z = 760.62 [M-H] ^+ ^and 798.58 [M-K^+^]) were found to be overexpressed in breast cancer. In our previous publication [24], we demonstrated that cholangiocarcinomas and pancreatic cancers have increased PC {34:1} (m/z = 760). We also validated the identity of the PC {34:1} peak by comparing MS/MS spectra of [M+H]^+ ^ion (m/z 760) of 34:1-PC species found on a cholangiocarcinoma sample with those of standard 18:1/16:0-PC species [24]. This peak was also increased in our ovarian cancer tissue samples as compared with adjacent normal tissue *(manuscript in preparation)*. Membrane phospholipid composition influences the activity of membrane-associated phospholipase A2 (PLA2) [25], which plays a role in carcinogenesis [26], and that overexpression of ChoKα1, which generates PC, is oncogenic [27].

More importantly, we have found that luminal, HER2+, and triple-negative breast cancer subtypes have different protein and lipid MALDI MS profiles. This result is consistent with a previous report that protein MALDI imaging MS classifies breast cancer tissue specimens according to HER2 status [13]. Using similar tissue MALDI MS technology, we confirmed and extended previous reports by identifying the intrinsic subtype-specific protein and lipid profiles. Brozkova, *et al*. also reported that SELDI-TOF MS could classify breast cancer tissue lysates to five groups which were analogous to intrinsic subtypes [28]. Thus, our MALDI profiles contain information about histopathological and biological feature of this heterogeneous disease [29]. Molecular profiles we identified are consistent with previous data generated via different analysis platforms. We have found that triple-negative breast cancers overexpress PC {32:1} [M+H]^+ ^(m/z = 732.58) and PC {30:0} [M+H]^+ ^(m/z = 706.5), compared with luminal and HER2 subtypes. According to a recent report by Hilvo *et al*., these lipids are underexpressed in ER-positive breast cancer tissue samples, compared with ER-negative samples (*P *= 0.001 and *P *< 0.001, respectively) [16]. In our data, these lipids were also positively correlated with ER negativity (*P *= 0.029 and 0.002, respectively).

Notably, our relatively simple approach takes less analytic time and tissue amount than lysate-based proteomics technologies used by aforementioned studies [16,29], which provides a clear advantage as a possible diagnostic aid, once our discriminatory profiles are validated in a larger dataset. Our novel two-matrix (SA and DHB/CHCA) MALDI profiling offers additional layer of molecular information, and, therefore, may possibly capture biological features of the breast cancer better than one-matrix MALDI profiling. Conversely, our relatively simple approach needs to be improved for obtaining tumor cell-specific mass spectra to better profile complex clinical material such as the needle biopsy of breast cancers, by using MALDI imaging MS [13] or a robotic spotter that deposits micron-sized droplets of matrix [12]. Also, our study is limited by incomplete assignment of discriminatory peaks using MS/MS, and by the sample size which is sufficient for statistical significance yet relatively small. Further studies on a larger scale are clearly warranted to validate discriminatory protein and lipid profiles identified by this proof-of-concept study.

## Conclusions

Luminal, HER2+, and triple-negative breast cancer subtypes have different protein and lipid MALDI MS profiles. Protein and lipid profiles identified by our novel two-matrix (SA and DHB/CHCA) MALDI analyses are sensitive and may capture biological features of the breast cancer.

## Competing interests

The authors declare that they have no competing interests.

## Authors' contributions

HSK designed experiments and collected samples; SCL processed tissue and performed MALDI MS; YSP performed MSMS; YEJ performed MALDI MS; JHL performed MALDI MS; SYJ collected samples; IHP collected samples; SHJ collected samples; HMP analyzed clinical data; CWY performed histopathological review; SHP reviewed manuscript; SYH performed MALDI MS; KPK performed MALDI MS; WHK performed MSMS; JSR designed experiments, performed clinical annotation, and contributed to the writing of the manuscript. HKK designed experiments and analyzed the data. All authors read and approved the final manuscript.

## Pre-publication history

The pre-publication history for this paper can be accessed here:

http://www.biomedcentral.com/1471-2407/11/465/prepub

## Supplementary Material

Additional file 1 **Procedures for MALDI MS analysis**. A representative cancer tissue cryosection, with the DHB/CHCA matrix applied at the locations marked in the H&E section, is shown at the bottom. The H&E section marked at discrete locations (enriched in tumor cells) using a red color marker pen (*center*) and magnified (*× *20) areas of the H&E-stained section corresponding to matrix-loaded spots (*top left and right*) are shown at the top. Click here for file
