# Supplementary material for: Protein and lipid MALDI profiles classify breast cancers according to the intrinsic subtype
Source: BMC Cancer. 2011 Oct 27;11:465. doi: 10.1186/1471-2407-11-465 (PMC3218066; doi:10.1186/1471-2407-11-465)
Supplement: Additional file 1 — Procedures for MALDI MS analysis. A representative cancer tissue cryosection, with the DHB/CHCA matrix applied at the locations marked in the H&E section, is shown at the bottom. The H&E section marked at discrete locations (enriched in tumor cells) using a red color marker pen (center) and magnified (× 20) areas of the H&E-stained section corresponding to matrix-loaded spots (top left and right) are shown at the top. [file 1471-2407-11-465-S1.DOC]

**Protein and lipid MALDI profiles classify breast cancers according to their intrinsic subtype.**

**Supplementary Information**

**Supplementary Figure**

Fig 1S. Procedures for MALDI MS analysis. A representative cancer tissue cryosection, with the DHB/CHCA matrix applied at the locations marked in the H&E section, is shown at the bottom. The H&E section marked at discrete locations (enriched in tumor cells) using a red color marker pen (*center*) and magnified (*×* 20) areas of the H&E-stained section corresponding to matrix-loaded spots (*top left and right*) are shown at the top.

**
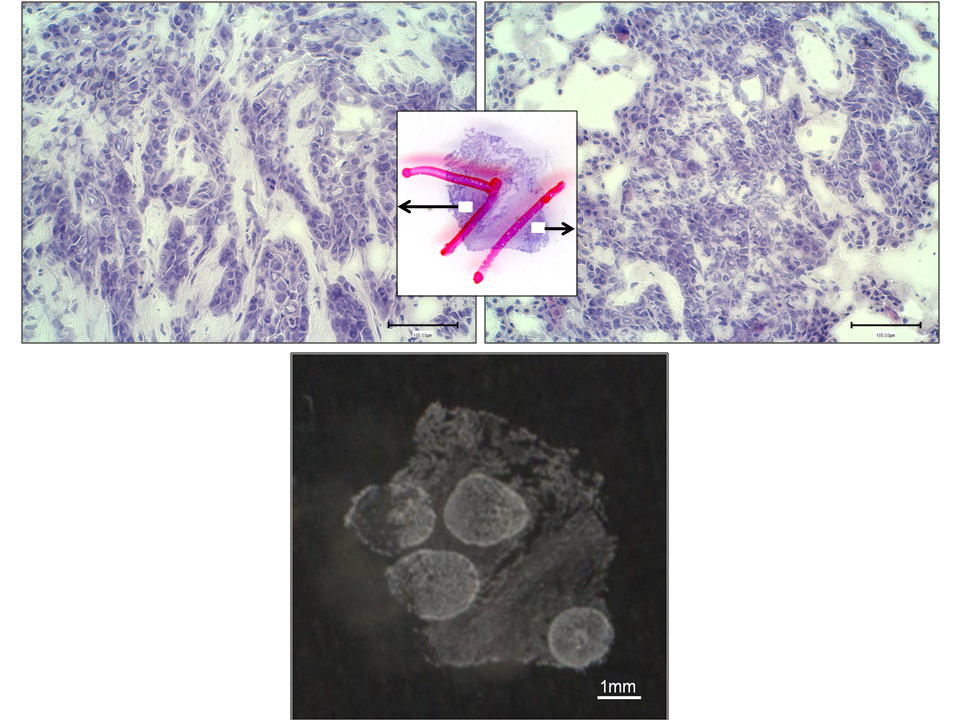
**
